# Development of Unidirectional Cellular Structure with Multiple Pipe Layers and Characterisation of Its Mechanical Properties

**DOI:** 10.3390/ma13173880

**Published:** 2020-09-02

**Authors:** Matej Vesenjak, Yutaka Nakashima, Kazuyuki Hokamoto, Zoran Ren, Yasuo Marumo

**Affiliations:** 1 Faculty of Mechanical Engineering, University of Maribor, 2000 Maribor, Slovenia; zoran.ren@um.si; 2 Graduate School of Science and Technology, Kumamoto University, Kumamoto 860-8555, Japan; 189d8521@st.kumamoto-u.ac.jp; 3 Institute of Industrial Nanomaterials, Kumamoto University, Kumamoto 860-8555, Japan; hokamoto@mech.kumamoto-u.ac.jp; 4 International Research Organization for Advanced Science and Technology, Kumamoto University, Kumamoto 860-8555, Japan; 5 Faculty of Advanced Science and Technology, Kumamoto University, Kumamoto 860-8555, Japan; marumo@mech.kumamoto-u.ac.jp

**Keywords:** cellular structure, unidirectional cells, UniPore, multiple pipe layers, copper, fabrication, explosive welding, metallography, mechanical properties

## Abstract

This study is concerned with the development of a new unidirectional cellular (UniPore) copper structure with multiple concentric pipe layers. The investigated UniPore structures were grouped into three main types, each having a different number of pipes (3, 4, and 5 pipes per transversal cross-section) and different pore arrangements. The specimens were fabricated by explosive compaction to achieve tightly compacted structures with a quasi-constant cross-section along the length of the specimens. The bonding between copper pipes was observed by a metallographic investigation, which showed that the pipes and bars were compressed tightly without voids. However, they were not welded together. The mechanical properties were determined by quasi-static compressive testing, where the typical behaviour for cellular materials was noted. The study showed that porosity significantly influences the mechanical properties, even more so than the arrangement of the pipes.

## 1. Introduction

Cellular metals are some of the most suitable materials for modern lightweight engineering structures due to their mechanical and thermal properties, which result in diverse functionalities. Ashby et al. [1] described the properties of cellular metals in detail, which depend mainly on the base material, porosity, morphology, topology, and fabrication procedure. With recent developments in the fabrication processes, the properties of the cellular metal can be precisely engineered to adapt to the specific needs of each application [2]. An internal cellular structure with a repeatable and ordered topology is preferred to assure a high level of mechanical and thermal response repeatability. The cellular metal structures can be classified into four categories, ranging from the least control over the cell shape, size, and topology up to full geometry control [3]: (i) stochastic (fully irregular internal structure, e.g., open- and closed-cell foams), (ii) partially ordered (enhanced structural control at the cell level, e.g., syntactic foams, advanced pore morphology foam), (iii) ordered (regular and ordered assembly of identical structural elements, e.g., Kagome and UniPore structures), and (iv) designed-for-purpose (structures based on additive manufacturing, e.g., auxetic structures [4,5,6]). Cellular metals have been used in various applications due to their multifunctionality, including as impact energy absorbers, filters, heat exchangers, implants, and insulators. However, to increase their applicability and cost-efficiency, more knowledge about these complex structures should be obtained in terms of their alloy development, foam homogeneity, and process development according to Garcia-Moreno [7].

The idea of unidirectional cellular metals was initiated by the development of Gasar structures based on the metal–gas eutectic reaction by Shapovalov [8]. These structures offered tailored geometries (i.e., high level of regularity in their topology and morphology, cell size variation), improved mechanical properties in terms of their strength and rigidity, and flexibility in terms of their permeability, while their fabrication was simple and cheap. A similar type of cellular metal, the unidirectional lotus-type cellular metals based on unidirectional solidification, was proposed by Nakajima [9]. The geometrical, mechanical, and thermal properties of these metals were extensively studied. The influence of the porosity and pore size variation on the mechanical properties (e.g., elastic modulus) was analysed by Kovačič et al. [10] computationally and experimentally based on image recognition and statistical study of the pore morphology. The study confirmed the random spatial distribution of the pores and that accurate computational results can be obtained based on indirect reconstruction. The mechanical properties of lotus-type porous metal were analysed by Kujime et al. [11], utilizing uniaxial tests. They studied the yield and ultimate tensile strength and concluded that an increase in the strength can be attributed to the precipitation strengthening. The effective thermal conductivity, elastic modulus, and yield strength of lotus-type structures were also determined by Fiedler et al. [12]. Additionally, the strong anisotropy of the material was taken into account. The thermal anisotropy was studied using numerical and analytical approaches. It was found that the lotus-type porous structure enables directional thermal conduction and efficient transportation of the thermal energy.

The UniPore structures were proposed by Hokamoto [13] to avoid geometrical shortcomings of Gasar and lotus-type cellular metals. The UniPore structures provide for very long parallel cells, a nearly constant cross-section throughout the length, constant wall thickness, constant cell size, and complete isolation between cells. The fabrication procedure for UniPore structures is based on the explosive compaction and explosive welding process [14] of an outer pipe densely filled with smaller inner pipes. The inner pipes are filled with a low-melting-point material (e.g., paraffin) to avoid complete densification upon radial explosive compaction. The influence of the fabrication process on the mechanical properties of the base material was extensively studied by Vesenjak et al. [15] based on uniaxial experimental testing supported by direct image correlation (to precisely follow the displacement and strain fields). It was shown that explosive treatment significantly influences the global mechanical response throughout the complete loading range. The fabrication process has also been analysed by computational simulations [16]. The study revealed that during the fabrication, the velocity of the outer pipe is of significant importance. If the velocity is inadequate, the walls of the inner pipes have the potential to fail, which depends on their initial position and thickness. The initial UniPore structures were fabricated from copper [17]. The detailed metallographic analysis showed effective compaction at different porosities, with nearly constant cross-sections and predictable compressive properties (with low deviation). Furthermore, ductile failure and excellent energy absorption capacity were observed. UniPore structures made of aluminium were also developed [18]. Again, the fabrication procedure proved to be very effective, with only a few fractured inner pipes, which can be additionally tuned through proper selection of the pipe thickness. The dynamic behaviour of UniPore structures has been described by Nishi et al. [19]. UniPore specimens were subjected to Taylor impact testing using a one-stage powder gun. During the high-velocity impact, the metal jet and high-stress concentrations positioned near to the pores were observed, which were also confirmed by computational results. The possibility of using the UniPore structures as thermal capacitors was studied by Fiedler et al. [20]. For that purpose, transient thermal experimental tests were performed to stabilise the temperature of the heat source. The study showed that thermal conduction is the primary heat transfer mechanism. Furthermore, the UniPore specimens successfully decreased the temperature of the heat source and proved to be effective thermal capacitors. To simplify the fabrication procedure and to reduce the production costs, the rolled UniPore structure fabrication procedure was developed by using cheap thin copper sheets with uniformly positioned spacers (bars) made of low-melting-point material (e.g., acryl) and radial explosive compaction [21].

Utsunomiya et al. [22] proposed another approach to fabricate a two-dimensional cellular copper structure with a constant cross-section and excellent mechanical properties. This structure was based on cold extrusion (plastic metalworking process with solid-phase bonding [23]) in three steps: (i) setting up an assembly of copper and aluminium wires, (ii) bonding by extrusion, and (iii) chemical leaching of aluminium. However, only limited lengths can be fabricated using this technology, since an extremely long leaching period is required. Additionally, special care has to be taken during extrusion to avoid necking at lower thicknesses and gaps between the initial assembly of wires [24].

This study is concerned with the development of a unidirectional cellular metal structure (UniPore) with multiple pipe layers and longitudinal cells and characterisation of its mechanical properties. The study takes advantage of the UniPore structure’s fabrication method and initial concentric assembly of wires (bars and pipes) as fabricated by Utsunomiya, which are described above. The leaching step is minimised by substituting the aluminium wires with polymer bars. The UniPore fabrication method enables fabrication of longer specimens in the order of several metres with minimal changes in the pipe wall thickness due to radial compaction. The newly developed specimens, using an upgraded UniPore structure fabrication method, were thoroughly analysed by metallographic analysis, while their mechanical properties and deformation mechanism were characterised with compressive experimental tests.

## 2. Materials and Fabrication of the Specimens

The fabrication procedure for unidirectional cellular metal structures with longitudinal cells (UniPore) consists of the following steps [25]:

1. Preparation of the specimen in the form of an assembly of metal pipes or bars, where the voids are filled with low-melting-point material (i.e., paraffin, polymer);
2. Placement into the explosive container, where the specimen is surrounded by the primary explosive;
3. Explosive compaction, with the main explosive detonated by the booster;
4. Removal of the low-melting-point material by thermal treatment.

**Figure 1:**
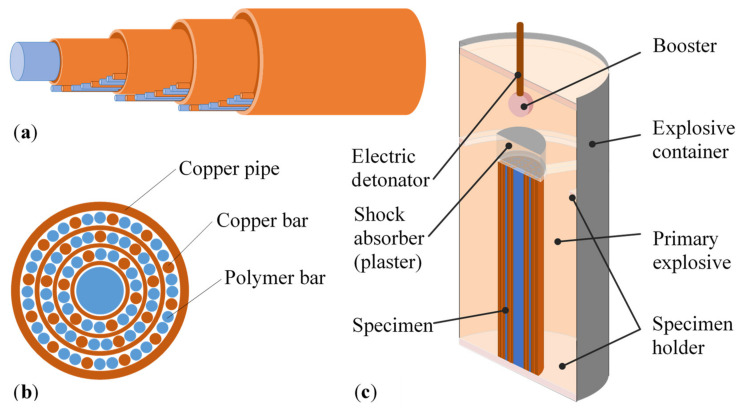
Specimen preparation: (**a**) specimen assembly, (**b**) cross-section of the assembly, and (**c**) experimental setup [25].

**Figure 2:**
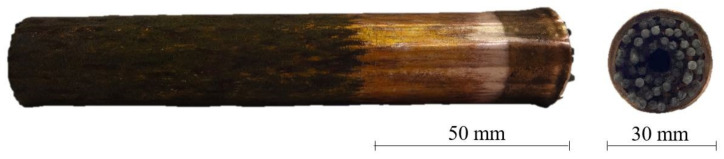
UniPore specimen after the explosive compaction.

The specimens were assembled with concentric layers of copper pipes, where the distance between the pipes was controlled by layers of copper and polymer bars (Figure 1 and Figure 2). Polymer bars were used as fillers in voids to avoid full densification during the explosive compaction. They were removed by thermal treatment after the explosive compaction.

Copper (JIS-C1220) pipes with different diameters and wall thicknesses were used in this study to fabricate specimens with different cross-section arrangements, varying wall thicknesses, and varying levels of porosity of the final UniPore structure. Layers of copper and polymer bars were placed between the pipes. The solid copper (JIS-C1220) bars measured 2 mm in diameter, while the polymer filler (acryl or glue sticks) bars measured 2 mm, 8 mm, or 14 mm in diameter. The length of the specimens was 100 mm. Three types of UniPore structures were fabricated, with differing numbers of pipes: type 1 contained 5 concentric copper pipes, type 2 contained 4 concentric copper pipes, and type 3 contained 3 concentric copper pipes. Two different assemblies of copper and polymer bars were prepared for each specimen type. The numbers of layers, bars, and pipes and the actual dimensions of the prepared assemblies are listed in Table 1.

Each specimen assembly was positioned centrally in the explosive container made of polymethyl methacrylate (PMMA; known as acrylic glass or plexiglass), which measured 165 mm in height and 83 mm in diameter (Figure 1c), surrounded by 450 g of the ammonium-nitrate-based primary explosive ANFO-A. The detonation of the primary explosive was initiated by an electric detonator with a 3 g SEP (65% wt. pentaerythritol tetranitrate (PETN) and 35% wt. paraffin) booster. This resulted in a radial high-pressure (approximately 0.9 GPa; data obtained from analysis on explosive compaction of cellular metals provided by Hokamoto et al. [25]) wave propagating along the length of the specimen, which completely compacted the specimen and consequently explosively welded the pipes, as represented by the wavy interface between the pipe surfaces observed by Meyers and Murr [26]. The recovered compacted specimens (Figure 2) were then submerged in the acetone bath for approximately 48 h to remove the polymer filler and obtain the final copper UniPore specimens with longitudinal cells.

**Figure 3:**
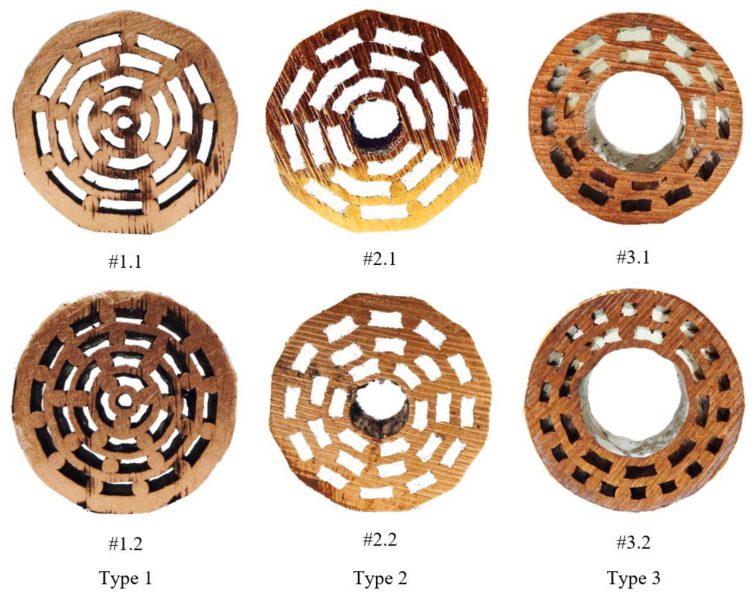
Cross-sections of fabricated specimens.

The UniPore specimens were then cut into smaller specimens suitable for metallography and mechanical testing. The cross-sections with different topologies of obtained specimens with respect to their initial assemblies (Table 1) are shown in Figure 3.

**Figure 4:**
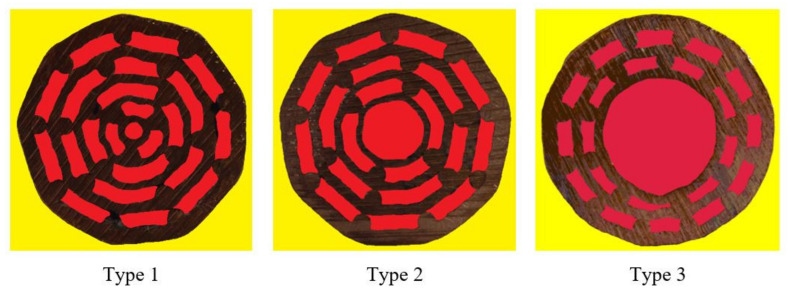
Evaluation of the actual porosity utilizing digital image correlation.

The cross-sections of the fabricated specimens were also visually analysed. It was observed that the cross-sections did not vary through the length of the specimens, assuring a constant cross-section along the length of the specimens, with a constant wall thickness and cell size and shape. The porosity of the specimens was evaluated before (theoretical porosity) and after (actual porosity) explosive compaction of the specimens. The theoretical porosity was calculated as the ratio between the cross-sectional area of polymer bars and the cross-sectional area of copper pipes, copper bars, and polymer bars. In contrast, the actual porosity was determined by digital image correlation of the specimens’ cross-sections (Figure 4). The number of red pixels divided by the number of brown pixels (number of yellow pixels subtracted from the total number of pixels) represents the actual porosity of the specimens. The theoretical and actual porosity are listed in Table 1. Due to the heavy compaction at high pressure, the actual porosity was slightly lower than the theoretical one.

The porosity of fabricated specimens varied between 32% and 49%. The lowest porosity was observed for type 1 specimens, while the highest porosity was measured for type 3 specimens. For comparison, the porosity values of previous copper [17] and aluminium [18] UniPore structures were between 39% and 58% and between 15% and 52%, respectively. The porosity values of the rolled copper [21] and rolled aluminium [27] UniPore structures were between 31% and 42% and between 9% and 18%, respectively. The porosity range of the specimens fabricated by cold extrusion bonding was between 14% and 54% [22].

## 3. Methods

### 3.1. Metallography

The metallographic analysis of the UniPore structures with multiple pipe layers was performed using the Nikon UM-2 light microscope device (Nikon, Tokyo, Japan). The standard metallographic methods (embedment into the epoxy resin, grinding, polishing, and chemical etching) were applied to prepare the samples. The transversal (for types 1, 2, and 3 specimens) and longitudinal (only type 1 specimens) cross-sections were microscopically investigated with a magnification factor of up to 50.

### 3.2. Compressive Testing

The deformation behaviour and mechanical properties of the fabricated UniPore specimens were evaluated by uniaxial quasi-static compressive testing using the servo-hydraulic Shimadzu AutoGraph IS-10T testing machine (Shimadzu, Kyoto, Japan). The UniPore specimens were cut perpendicular to the cell orientation (transversal cross-section) into cylindrical compressive specimens measuring 10 mm in thickness. Due to the expected repeatability (constant cross-section) of the results, only 2 specimens were prepared from each fabricated UniPore specimen. The specimens (12 in total) were subjected to quasi-static loading conditions with a cross-head rate of 5 mm/min. A thin polytetrafluoroethylene (PTFE—Teflon) layer was positioned between the specimens and the loading and support plates to reduce the friction. The displacement and force during compression were measured by the testing device, while the sample deformation behaviour was captured by a digital camera. The measured force–displacement relationship was used to determine the mechanical properties of the new UniPore structures (e.g., force plateau, energy absorption capability), while observation of the video recordings allowed for evaluation of the deformation mechanisms during the loading process. The force plateau was defined as the average force in the plateau region of the force–displacement diagram. The energy absorption was determined by integrating the force along with the displacement. The energy absorption efficiency was evaluated as the ratio between the actual and ideally absorbed energy.

## 4. Results of a Metallographic Investigation

The connectivity between the individual components (copper pipes and bars) of the UniPore structure depends on the colliding conditions during the fabrication process based on explosive compaction. The connectivity is related to the velocity and inclination angles of the copper pipes and bars required for explosive welding with tight bonding [14]. A metallographic analysis was performed on representative samples of the specimens to observe the metallic bonding between the surfaces of copper pipes and bars.

**Figure 5:**
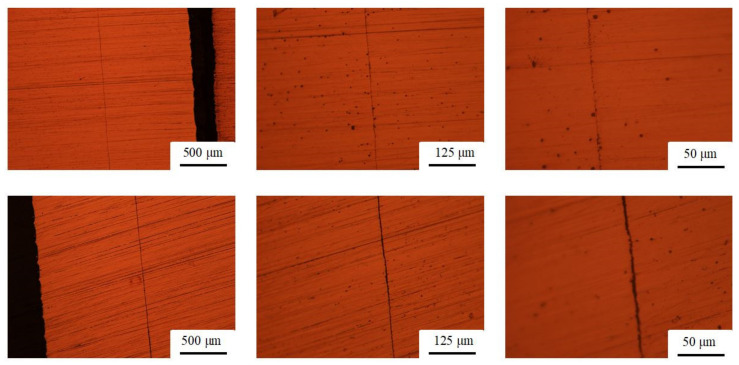
Metallographic images of longitudinal cross-sections (upper row: specimen #2.1; lower row: specimen #2.2).

**Figure 6:**
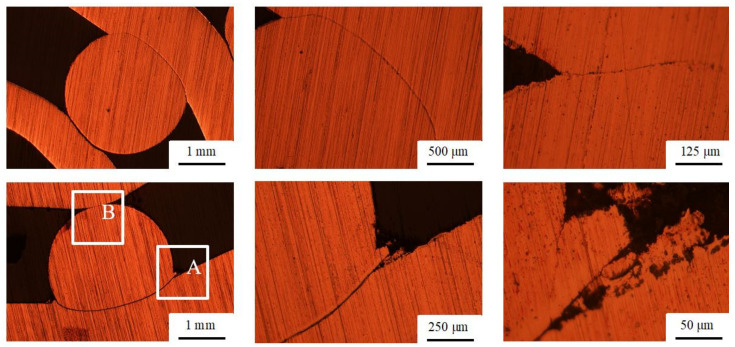
Metallographic images of transversal cross-sections (upper row: specimen #1.1, lower row: specimen #1.2).

The investigated segments of the longitudinal cross-sections are shown in Figure 6. A wavy interface, which is typical for strong interfacial bonding due to intense fluidisation of the metals, was not observed. From the top left image in Figure 5, it can be observed that the samples are tightly compressed without voids but not tightly bonded together, which might suggest a lower bonding strength.

**Figure 7:**
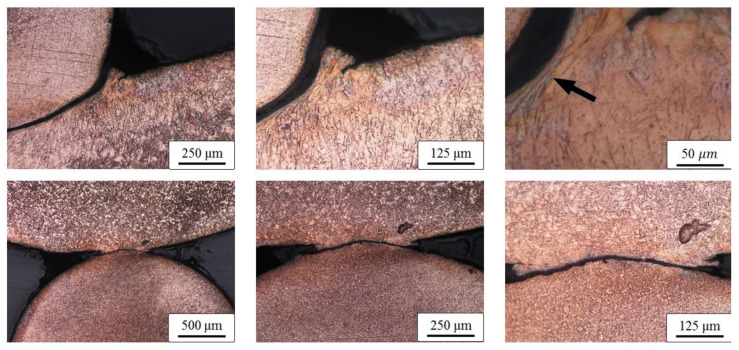
Metallographic images of transversal cross-sections (specimen #1.2): magnification of section A (Figure 6), upper row; magnification of section B (Figure 6), lower row.

The transversal cross-section segments of the specimens are shown in Figure 6. Large deformation of the copper bar due to pipe collision is evident. However, the deformation at the contact point, where the bar collided with the inner pipe, is not significant. A small deformed zone spreading sidewise can be observed in section A (Figure 6). Both contact sections A and B were additionally etched and are shown in Figure 7. Although the image does not precisely represent the same position, it is possible to observe that the contact surfaces are deformed or spread sidewise, causing an intense deformation of each crystalline grain in the region denoted with an arrow. The phenomenon is similar to the formation of the metal jet typically caused in explosive welding [14]. However, it is disrupted (discontinued) due to the presence of the neighboring polymer material (polymer bars placed next to the copper bar).

## 5. Results of Compressive Testing

The UniPore structures can also be used as structural load-carrying components. Thus, it is vital to understand and characterise their deformation and collapse mechanisms and their responses when subjected to mechanical loading. The uniaxial quasi-static compressive tests in the transversal direction of the fabricated specimens were performed for this purpose.

### 5.1. Type 1 Specimens

**Figure 8:**
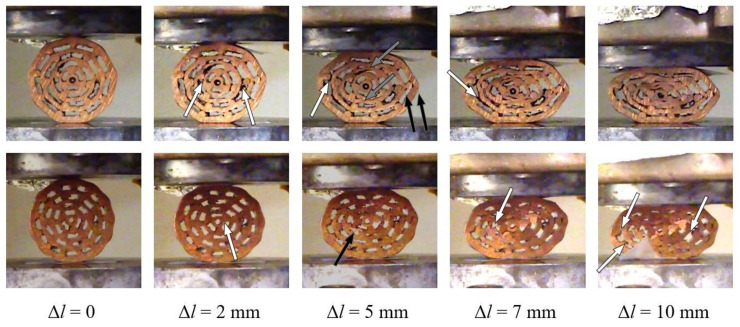
Deformation mechanism (white arrows denote debonding, black denote bending or buckling, and grey arrows denote contact) of the type 1 specimens (#1.1: upper row; #1.2: lower row).

Type 1 specimens represent the group with the lowest average porosity. Their deformation behaviour is shown in Figure 8.

Uniform deformation throughout the loading process was observed for both specimens. Some intermetallic bonds between bars and pipe surfaces failed in the initial loading phase of specimen #1.1. It is interesting to notice that the bonding failure appears on the outer surface in all three cases. The surfaces of two inner pipes come into contact almost simultaneously at a displacement of 5 mm. Additionally, the outer two copper pipes begin to buckle, which is even more pronounced with larger displacements. In contrast, specimen #1.2 (with a slightly lower porosity) experienced separation of the bonded surfaces throughout the loading process. Contact between pipe surfaces appeared at a displacement of 10 mm due to the lower porosity of the sample. However, local buckling of the inner pipe was observed at a displacement of 5 mm.

**Figure 9:**
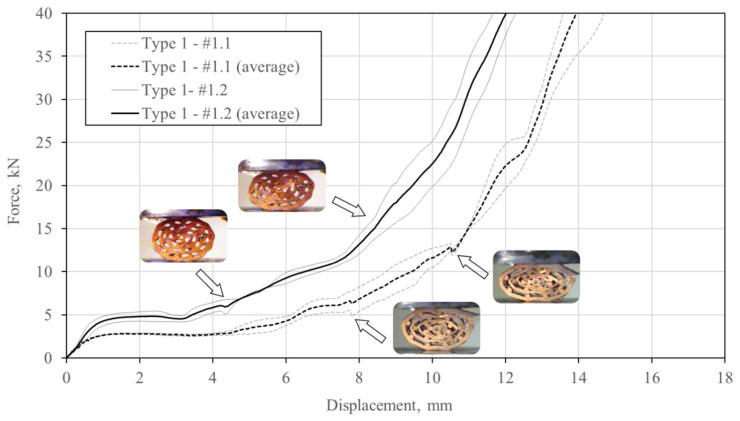
Compressive force–displacement relationship of the type 1 specimens.

The compressive force–displacement relationship of the type 1 specimens is shown in Figure 9, which shows the typical behaviour for cellular materials [1]—after a quasi-linear increase in stiffness, the force plateau is established until densification, when the stiffness increases. The force plateau is evident up to a displacement of approximately 4 mm. The stiffness gradually increases after this due to emerging contacts between the surfaces of the pipes. The stiffness and postponed densification of specimen #1.2 are related to the higher porosity of the sample.

### 5.2. Type 2 Specimens

**Figure 10:**
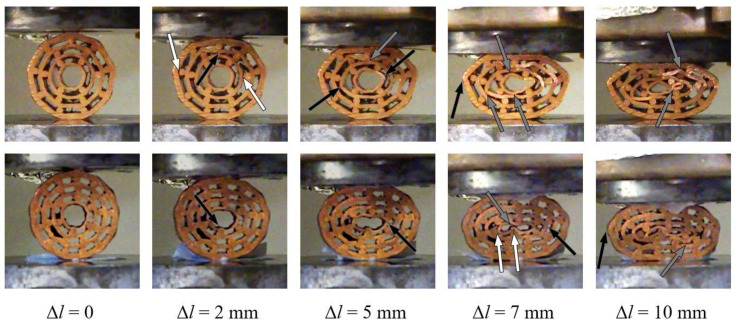
Deformation mechanism (white arrows denote debonding, black denote bending or buckling, and grey arrows denote contact) of the type 2 specimens (#2.1: upper row; #2.2: lower row).

The type 2 specimens have fewer pipes and a bigger hole in the centre of each specimen than the type 1 specimens. The average porosity of type 2 specimens is higher than of type 1 specimens. Their deformation mechanism can be observed in Figure 10. In general, lower rates of debonding and failure of the bonded interfaces was noted for both specimens (#2.1 and #2.2) in comparison to the type 1 specimens. The inner pipe began to bend at a displacement of 5 mm in the case of specimen #2.1. Upon further loading, the bending of the inner pipe resulted in point contact. All copper pipes began to buckle almost simultaneously after the displacement of 7 mm. Consequently, several concentrated contacts can be observed due to local bending and buckling of the pipes. In the case of specimen #2.2, the local bending of the inner pipe was initiated at a displacement of 5 mm. The same pipe then buckles, which results in a self-contact at the displacement of 7 mm. More dense distribution of the copper bars was the reason for the contact between the surfaces of inner pipes being less apparent and postponed in comparison to specimens #1.1 and #2.1.

**Figure 11:**
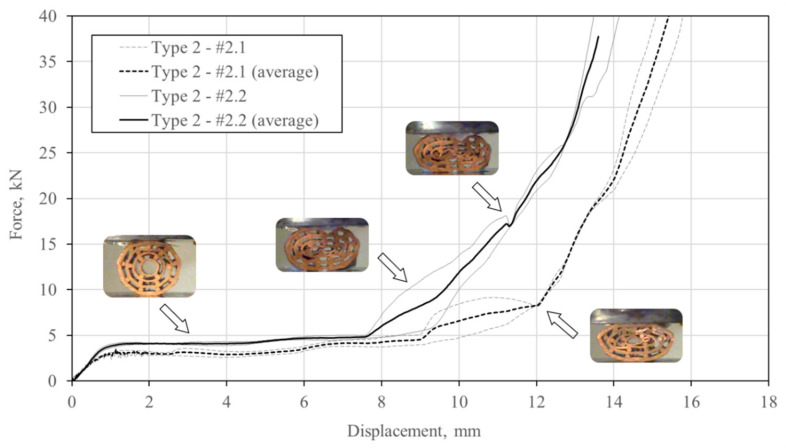
Compressive force–displacement relationship of the type 2 specimens.

The compressive force–displacement relationship of the type 2 specimens is shown in Figure 11. The arrangement of pipes assures an extended force plateau until a displacement of approximately 8 mm. The prolonged force plateau is a consequence of the lower number of pipes in type 2 specimens. The deviation between the specimen’s response is very low, except at the beginning of the densification due to slightly different deformation and failure mechanisms between single specimens.

### 5.3. Type 3 Specimens

**Figure 12:**
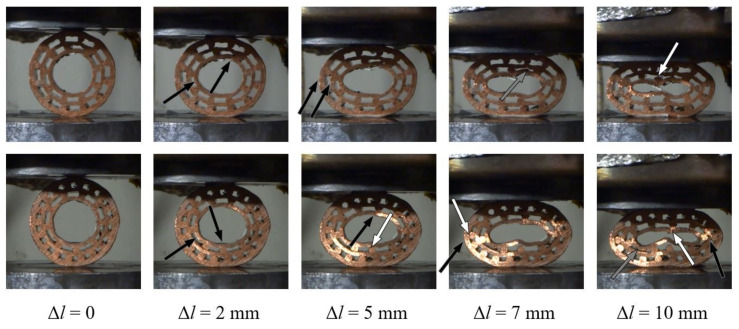
Deformation mechanism (white arrows denote debonding, black denote bending or buckling, and grey arrows denote contact) of the type 3 specimens (#3.1: upper row; #3.2: lower row).

The type 3 specimens have the least number of pipes and the biggest hole in the center of the specimen of all investigated specimens. Consequently, they have the highest average porosity. Their deformation sequence during the compressive loading is shown in Figure 12. Similarly to the deformation observation of the type 2 specimens, the type 3 specimens also experience less separation of initially bonded components in comparison to the type 1 specimens. This might be a result of the less tightly packed cross-sectional assembly of the copper pipes and bars. The inner pipe of specimen #3.1 begins to locally bend at the top around the above bonded bar and buckles on its left side at a displacement of 2 mm. The buckling of the outer to pipes was also observed with loading increase at a displacement of 5 mm.

A single contact between the surfaces of two inner pipes was observed at a displacement of 7 mm. A very similar deformation behaviour (local bending due to the bonded bars below and above and buckling) of the inner pipe at displacement lengths of 2 mm and 5 mm can be observed in the case of specimen #3.2. The local bending (and later buckling) results in the debonding between the pipe and the bar. The dense and regular arrangement of bars again prevents contact between pipes until a displacement of 7 mm.

**Figure 13:**
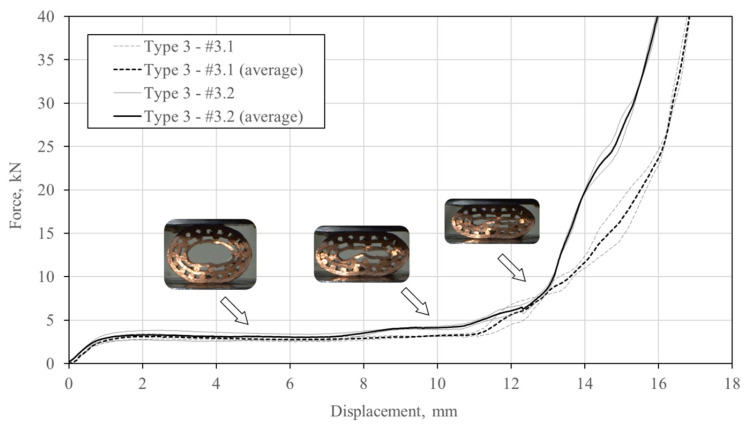
Compressive force–displacement relationship of the type 3 specimens.

The compressive force–displacement relationship of the type 3 specimens is represented in Figure 13. It can be observed that the constant force plateau is prolonged until a displacement of approximately 11 mm. The densification is initiated by the self-contact of the inner pipe surface. The deviation between single responses is almost negligible, which makes this type of specimens less sensitive to the fabrication defects.

## 6. Comparison

**Figure 14:**
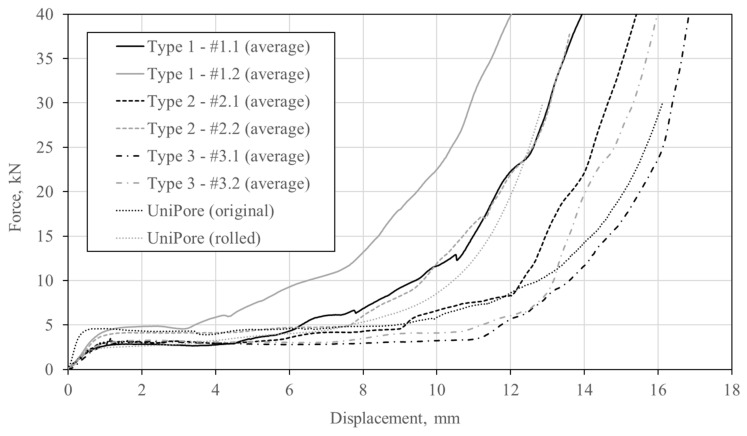
Comparison of compressive responses.

Figure 14 shows the average compressive force–displacement relationships of tested UniPore specimens. The responses of the originally developed UniPore structure [17] and rolled copper UniPore structure [21] are added for comparison purposes. The porosities of the original and rolled UniPore structures are 0.49 and 0.42, respectively. It can be observed for all types of structures that a constant force plateau follows the initial quasi-linear stiffness increase. The type 1 specimens exhibit the shortest force plateau, while the type 3 specimens the most prolonged force plateau. It can also be observed that the response of the original UniPore structures is similar to the response of the type 3 specimens, while the response of the rolled UniPore structures shows early densification, which is comparable to the responses of type 1 and type 3 specimens. The metallographic investigation of specimen types 1, 2, and 3 showed that the samples were tightly compressed but not bonded together (Figure 5, Figure 6 and Figure 7), which resulted in lower bonding strengths but also in smoother load–displacement curves, without significant interfacial sliding or abrupt stiffness degradation.

The observed force plateau and calculated energy absorption values of the analysed specimens are listed in Table 2. The force plateau was evaluated as the average force between displacements of 2 and 4 mm. At the same time, the energy absorption represents the integrated values of force over displacement from 0 up to 5 and 10 mm, respectively.

The porosity significantly influenced the mechanical properties, even more so than the arrangement of the pipes. The specimens with the lowest porosity values (e.g., #1.2 and #2.2) gave the highest force plateau values, which were comparable to the force plateau value of the original UniPore structure. Consequently, their energy absorption capacity values were also the highest.

## 7. Conclusions

The study describes the development of a new unidirectional cellular copper structure with concentric multiple pipe layers. The specimens were fabricated by explosive compaction to achieve bonding between single pipe components. Different arrangements of inner pipes and layers (types 1, 2, and 3) were analysed experimentally. The bonding between copper pipes was observed by a metallographic investigation, which showed that the pipes and bars were tightly compressed without voids. However, they were not welded together. The mechanical response was analysed by quasi-static compressive tests, where typical behaviour for cellular materials was noted. The specimens with the highest numbers of layers and lowest porosity values (type 1) showed the shortest force plateaus, while the specimens with the lowest numbers of layers and highest porosity values (type 3) exhibited the most prolonged constant force plateaus. The study has shown that porosity significantly influences the mechanical properties, even more so than the arrangement of pipes. The specimens with the lowest porosity values (e.g., #1.2 and #2.2) provided the highest force plateau values, which were comparable to the force plateau of the original UniPore structure. Consequently, their energy absorption capacity values were also the highest. The observation of the deformation mechanism provided an insight into the debonding process, bending and buckling of pipe walls, and contact between constituents, which is directly connected to the initiation of the densification.

## Figures and Tables

**Table 1:** Geometrical properties of the fabricated specimens.

Layer No.	Layer Material	Type 1		Type 2		Type 3	
		#1.1	#1.2	#2.1	#2.2	#3.1	#3.2
1	Cu pipe (*d_o_*/*d_i_*/*t*)	30/27/1.5	30/27/1.5	30/27/1.5	30/27/1.5	30/27/1.5	30/27/1.5
2	No. of Cu (acryl) bars	9 (27)	12 (24)	9 (27)	12 (24)	12 (24)	18 (18)
3	Cu pipe (*d_o_*/*d_i_*/*t*)	22/20/1	22/20/1	22/20/1	22/20/1	22/20/1	22/20/1
4	No. of Cu (acryl) bars	6 (21)	9 (18)	6 (21)	9 (18)	9 (18)	9 (18)
5	Cu pipe (*d_o_*/*d_i_*/*t*)	16/14/1	16/14/1	16/14/1	16/14/1	16/14/1	16/14/1
6	No. of Cu (acryl) bars	4 (14)	6 (12)	4 (14)	6 (12)	0 (1)	0 (1)
7	Cu pipe (*d_o_*/*d_i_*/*t*)	10/8/1	10/8/1	10/8/1	10/8/1	-	-
8	No. of Cu (acryl) bars	3 (6)	3 (6)	0 (1)	0 (1)	-	-
9	Cu pipe (*d_o_*/*d_i_*/*t*)	4/2/1	4/2/1	-	-	-	-
10	No. of Cu (acryl) bars	0 (1)	0 (1)	-	-	-	-
	Theoretical porosity [%]	37.7	32.2	41.5	36.5	49.3	44.3
	Actual porosity [%]	36.5	30.3	40.4	35.9	44.4	43.2

Remark: layer no.—a consecutive number of layers, starting with the outer layer towards the centre of the specimen; *d_o_* (mm)—outer diameter; *d_i_* (mm)—inner diameter; *t* (mm)—wall thickness.

**Table 2:** Mechanical properties of the UniPore specimens.

	Type 1		Type 2		Type 3		UniPore	UniPore
	#1.1	#1.2	#2.1	#2.2	#3.1	#3.2	Original	Rolled
Porosity (%)	36.5	30.3	40.4	35.9	44.4	43.2	49	42
Force plateau (kN)	2.74	4.96	3.05	4.11	3.06	3.20	4.19	2.92
Energy absorption up to displacement of 5 mm (J)	13.05	23.52	14.04	18.69	13.50	14.46	20.91	13.71
Energy absorption up to displacement of 10 mm (J)	46.67	88.90	35.63	49.84	28.12	31.59	44.98	40.06
Densification (mm)	4.9	3.4	11.9	8.9	11.3	10.4	11.5	8.5
